# Development and Characterization of a Luminescence-Based High-Throughput Serum Bactericidal Assay (L-SBA) to Assess Bactericidal Activity of Human Sera against Nontyphoidal *Salmonella*

**DOI:** 10.3390/mps5060100

**Published:** 2022-12-16

**Authors:** Maria Grazia Aruta, Daniele De Simone, Helen Dale, Esmelda Chirwa, Innocent Kadwala, Maurice Mbewe, Happy Banda, Melita Gordon, Mariagrazia Pizza, Francesco Berlanda Scorza, Tonney Nyirenda, Rocío Canals, Omar Rossi, on behalf of the Vacc-iNTS Consortium Collaborators

**Affiliations:** 1 GSK Vaccines Institute for Global Health Srl, Via Fiorentina 1, 53100 Siena, Italy; 2 Malawi Liverpool Wellcome Trust Programme, Queen Elizabeth Central Hospital, College of Medicine, Blantyre 30096, Malawi; 3 Pathology Department, Kamuzu University of Health Sciences, Blantyre 312225, Malawi; 4 Institute of Infection, Veterinary and Ecological Sciences, University of Liverpool, Liverpool L69 3BX, UK

**Keywords:** generalized modules for membrane antigens (GMMA), serum bactericidal assay (SBA), nontyphoidal *Salmonella*, functional assay, human sera, antibodies, vaccine, iNTS

## Abstract

*Salmonella* Typhimurium and *Salmonella* Enteritidis are leading causative agents of invasive nontyphoidal *Salmonella* (iNTS) disease, which represents one of the major causes of death and morbidity in sub-Saharan Africa, still partially underestimated. Large sero-epidemiological studies are necessary to unravel the burden of disease and guide the introduction of vaccines that are not yet available. Even if no correlate of protection has been determined so far for iNTS, the evaluation of complement-mediated functionality of antibodies generated towards natural infection or elicited upon vaccination may represent a big step towards this achievement. Here we present the setup and the intra-laboratory characterization in terms of repeatability, intermediate precision, linearity, and specificity of a high-throughput luminescence-based serum bactericidal assay (L-SBA). This method could be useful to perform sero-epidemiological studies across iNTS endemic countries and for evaluation of antibodies raised against iNTS vaccine candidates in upcoming clinical trials.

## 1. Introduction

Nontyphoidal *Salmonella* (NTS) is one of the leading causes of morbidity and mortality caused by enteric pathogens worldwide in both children and adults [1]. NTS serovars usually cause a self-limiting diarrheal illness in immunocompetent individuals in developed countries, while they are responsible for invasive NTS (iNTS) disease in sub-Saharan Africa [2]. In 2017, of an estimated 535,000 cases of iNTS infections in the world, 421,600 were estimated to have occurred in sub-Saharan Africa, with 77,500 associated deaths [3]. *Salmonella enterica* serovars Typhimurium (*S*. Typhimurium) and Enteritidis (*S*. Enteritidis) account for more than 90% of iNTS infections [4]. Malaria, malnutrition, and HIV are reported risk factors of iNTS disease [5,6,7,8,9,10,11,12]. Although the sources and modes of transmission of iNTS infections in developing countries are still uncertain, reported evidence supports healthy asymptomatic human carriers as the source or reservoir of transmission in households [13,14,15]. Increasing levels of multidrug resistance (MDR) in *Salmonella* species and reported extensively drug resistant (XDR) isolates make treatment with antibiotics difficult [16,17,18] and denote the urgency for introduction of more effective treatment strategies, such as vaccines [19,20]. No licensed vaccines to prevent iNTS disease are currently available [20]. There are only a few vaccine candidates under development, based either on traditional or non-conventional approaches to induce immunity against the O-antigen portion of the lipopolysaccharide. Among those, a bivalent formulation of *Salmonella* Typhimurium and *Salmonella* Enteritidis [4] generalized modules for membrane antigens (GMMA) has been proposed as a vaccine candidate against iNTS disease [21,22,23]. GMMA are extracellular vesicles, obtained in high yields by genetic manipulation, that show multiple bacterial antigens in their natural conformation, as they contain outer membrane lipids, outer membrane proteins, and soluble periplasmic components [24,25]. Being self-adjuvanted particles, GMMA represent an attractive and affordable technology to fight this disease in developing countries [23,26].

The pathogenesis of iNTS is complex, but the measurement of antigen-specific antibodies in vaccinated sera or in plasma from convalescent individuals is central to understanding the burden of disease and potential efficacy and impacts of vaccination [27]. However, the presence of antibodies in isolation is not sufficient to prove or disprove the ability of antibodies to kill the pathogen [27], and thus a proxy of functionality is necessary and could serve as a surrogate of protection. Several assays are in place to measure in vitro the functionality of antibodies elicited by vaccination, and among them, the serum bactericidal assay (SBA) represents the method of choice to determine the ability of antibodies to mediate complement-mediated killing of bacteria. This assay is well-established in the field of vaccinology [28] and is widely used in the *Salmonella* field [27]. Despite that, SBA has been rarely adopted for large epidemiological studies and/or clinical samples analysis because it is time-consuming and labor-intensive [28]. To overcome these bottlenecks, we developed a luminescence-based high-throughput SBA (L-SBA) in 96-well [29,30] and 384-well plate format [31]. The assay had been already characterized intra-laboratory in terms of specificity, linearity, and precision by using human-serum L-SBA [32]. Here we present the setup and the intra-laboratory characterization of the L-SBA assay using human sera from an endemic area, evaluating accuracy, repeatability, intermediate precision, linearity, and specificity, both against the *S*. Typhimurium and *S*. Enteritidis strains. We determined detection and quantification limits and demonstrated the high performance of the two assays, which are now ready to be used in different laboratories to perform large sero-epidemiological studies as well as to assess bactericidal activity of antibodies from upcoming clinical trials.

## 2. Materials and Methods

### 2.1. Bacterial Strains and Reagents

Working aliquots of *Salmonella enterica* serovar Typhimurium D23580 (clinical isolate from blood culture, Malawi) [11,33] and *Salmonella enterica* serovar Enteritidis CMCC4314 (corresponding to ATCC4931) were obtained from the Novartis Master Culture Collection (NMCC) [34], stored at −80 °C in 20% glycerol stocks, and grown overnight at 37 °C in Luria–Bertani (LB) medium, stirring at 180 rpm. The overnight bacterial suspensions were then diluted in fresh LB to an optical density at 600 nm (OD_600_) of 0.05 and incubated at 37 °C with 180 rpm agitation in an orbital shaker until they reached 0.20–0.25 OD_600_. Baby (3- to 4-week-old) rabbit complement (BRC purchased from Cederlane—CL3441-S100—Euroclone, Canada, at a final concentration of 50% for both assays) was stored in frozen aliquots and thawed immediately prior to use. Phosphate-buffered saline (PBS) at pH 7 was used for serum and bacterial dilutions [22]. 

GMMA with penta-acylated lipid A was purified from the *S*. Typhimurium and *S*. Enteritidis Δ*tolR*Δ*pagP*Δ*msbB* strains [22]. The O-antigen (OAg) was extracted from *S*. Typhimurium and *S*. Enteritidis GMMA by direct acid hydrolysis [35]. The O-antigens were fully characterized analytically in terms of sugar content, protein, and nucleic acid impurities as previously reported [21].

### 2.2. Serum Samples

The iNTS Primary Human Standard Serum was generated by the Malawi-Liverpool-Wellcome Trust Clinical Research Programme (MLW) by pooling sera from highly positive Malawian adult subjects who were originally enrolled in a community-based randomly selected cohort within the STRATAA (Strategic Typhoid Alliance across Africa and Asia) epidemiological study [36]. Subjects who contributed to the study donated about 100 mL of blood each. Sera was collected and screened to confirm positivity by *S*. Typhimurium and *S*. Enteritidis LPS in ELISA before being pooled. Working aliquots of the serum were stored at −80 °C until use. All samples tested in luminescence-based serum bactericidal assay (L-SBA) were previously heat inactivated (HI) at 56 °C for 30 min to remove endogenous complement activity. Various aliquots of HI iNTS Primary Human Standard Serum were used and treated as described below to determine the different assay parameters. 

Samples used to assess repeatability and intermediate precision: Each sample consisted of the same HI iNTS Primary Human Standard Serum; in total, 12 identical samples were assayed each day by two operators, and the assay was repeated on three different days by each of the two operators independently (with 72 samples in total, 36 per operator, and 12 on each day).

Samples to assess linearity: HI iNTS Primary Human Standard Serum was assayed neat or diluted at 1:4, 1:8, 1:16, 1:32, 1:64, and 1:128 with PBS prior to performing the assay.

Samples used to assess limit of detection and limit of quantification: HI iNTS Primary Human Standard Serum was diluted 100-fold in PBS prior to being assayed in SBA against *S*. Typhimurium and *S*. Enteritidis in order to generate a sample with low but detectable SBA titer (with the expected IC50 to be around 100). Twelve identical prediluted iNTS Primary Human Standard Serum samples were assayed on the same day by one operator. 

Samples to assess specificity: Two sets of samples were prepared to assess the homologous and heterologous specificity of the assay using the HI iNTS Primary Human Standard Serum diluted at 1:1 (*v*:*v*) in PBS alone and PBS supplemented with different quantities of homologous or heterologous purified OAg and GMMA. The HI iNTS Primary Human Standard Serum was spiked with homologous OAg and GMMA from *S*. Typhimurium and from *S*. Enteritidis at different final concentrations (3780 µg/mL and 5300 µg/mL as maximum OAg concentrations, respectively, for STm and SEn, in addition to 500, 200, 50, and 10 µg/mL OAg) and compared with a sample spiked at 1:1 (*v*:*v*) with PBS alone. Samples were incubated overnight (16–18 h) at 4 °C prior to being tested. Additionally, *S*. Typhimurium and *S*. Enteritidis GMMA were chosen as the homologous competitors at 10, 50, 200, and 500 µg/mL, respectively. The lowest concentration of homologous competitor among the ones tested able to inhibit ≥ 70% of the IC50 was subsequently used in a second experiment to determine the heterologous specificity. In the second experiment, the HI iNTS Primary Human Standard Serum diluted at 1:1 (*v*:*v*) in PBS was supplemented not only with homologous O-antigen (*S*. Typhimurium OAg in the case of STm homologous specificity and *S*. Enteritidis OAg in the case of SEn homologous specificity) to reconfirm the result previously obtained but also with heterologous O-antigens, represented by *S*. Typhimurium OAg in the case of SEn heterologous specificity and *S*. Enteritidis OAg in the case of STm heterologous specificity (heterologous but from the same species) or *Shigella flexneri* 3a OAg (heterologous from a different species). All the samples were prepared and assayed in comparison to samples preincubated overnight with an equal volume of PBS alone (undepleted) and were incubated overnight (16–18 h) at 4 °C prior to being tested.

### 2.3. Luminescence-Based SBA (L-SBA)

The luminescence-based serum bactericidal assay (L-SBA) was performed in 96-well round-bottom sterile plates (Corning). In each well of the SBA plate, different dilutions of HI test sera were added in the presence of an exogenous complement (BRC) and bacteria. The serial dilutions of HI sera (25 µL/well) were prepared in PBS directly in the SBA plate. The starting dilution of test serum in the assay was 1:100 (final dilution) to assess repeatability and intermediate precision, 1:400 (final dilution) to determine the limit of detection and limit of quantification, 1:4 (final dilution) for the linearity assessment, 1:1200 (final dilution) in the case of homologous specificity (final dilution), and 1:400 (final dilution) for heterologous specificity, followed by 3-fold dilution steps (up to 11 dilution points for the linearity evaluation and up to 7 dilution points for the other tests). One control well with no sera, which represents the control for non-specific complement killing and was also used for fitting purposes, was added on each serum dilution [32]. Using this format, up to 11 different test sera, plus a standard serum used to validate the assay, can be assayed on each 96-well plate. Log-phase cultures (OD600 = 0.22 ± 0.03) were prepared as described above and diluted to approximately 1 × 10^6^ colony-forming units (CFU)/mL in PBS. The luminescence at T0 was measured by diluting the appropriate volume of bacteria in 4 different replicates in PBS and mixing at 1:1 (*v*:*v*) with BacTiter-Glo Reagent (Promega, Southampton, UK) for 5 min at room temperature on an orbital shaker; the luminescent signal was detected by a luminometer (Synergy HT, Biotek, Swindon, UK).

Seventy-five µL/well of reaction mix constituted by target bacterial cells (10 µL/well), BRC (50 µL/well), and PBS medium (15 µL/well) were added to each well of the SBA plate containing HI serum dilutions (final reaction volume 100 µL), mixed, and incubated for 3 h at 37 °C. At the end of the incubation (T180), the SBA plate was centrifuged for 10 min at 4000× *g* at room temperature. The supernatant containing ATP derived from dead bacteria and SBA reagents was discarded, and the remaining live bacterial pellets were resuspended in PBS (100 µL/well), transferred in a white round-bottom 96-well plate (Greiner), and mixed at 1:1 (*v*:*v*) with BacTiter-Glo Reagent (Promega, Southampton, UK). The reaction was incubated for 5 min at room temperature on an orbital shaker, and the luminescent signal was detected by a luminometer (Synergy HT, Biotek, Swindon, UK).

### 2.4. Calculations

In the L-SBA readout, the level of luminescence detected is directly proportional to the number of living bacteria present in the wells, which is inversely proportional to the level of functional antibodies present in the serum. A 4-parameter non-linear regression was applied to the raw luminescence obtained for all the serum dilutions tested for each serum, assigning an arbitrary serum dilution of 10^15^ to the control well containing no sera. Fitting was performed, constraining the curves to have a bottom between 0 and below a value equal to the mean value of the luminescence detected at T180 for standard sera in wells in which bacteria are killed (i.e., the first three dilutions of 72 repeats of the standard tested for repeatability and intermediate precision evaluation) plus the standard deviation (SD) of luminescence detected. To validate the assay plate, the average luminescence at T180 detected in all wells with no sera had to be at least 10-fold and 2.5-fold higher (respectively, for *S*. Typhimurium L-SBA and *S*. Enteritidis L-SBA) with respect to the luminescence detected at T0. The dilution series were considered valid if the highest luminescence detected in each series at T180 was at least 0.6-fold the luminescence detected in the respective control well not containing sera. The results of the assay are expressed as the IC50, the serum dilution able to kill half of the bacteria present in the assay (thus the reciprocal serum dilution resulting in a 50% reduction of maximum luminescence.) IC50 calculations were made using the GraphPad Prism 7 software (GraphPad Software, La Jolla, CA, USA).

### 2.5. Statistical Analysis

A mixed-effects model considering the day and the operator as random and no fixed factors was used to estimate the repeatability (defined as the variability under the same operating conditions over a short interval of time), to estimate the intermediate precision (defined as the variability among different days and different operators), and to evaluate the contributions of the operator and day of analysis to the variability. The Minitab 18 software (Minitab Inc., Chicago, IL, USA) was used for statistical analysis, including the linearity assessment.

### 2.6. Ethical Statement

The human-serum pool used in this study was derived from Malawian healthy donors originally enrolled in the STRATAA epidemiological study [36]. The relevant ethical and regulatory approval was obtained from the respective institutional and national ethics review committees (National Health Sciences Research Committee approval # 15/11/1511). Written informed consent was obtained before enrollment from all subjects, and the trial was designed and conducted in accordance with the Good Clinical Practice Guidelines and the Declaration of Helsinki.

## 3. Results

To understand the ability of sera to kill the target pathogen, it is critical to measure vaccine efficacy and determine the burden of disease in the field. In order to establish a solid and accurate assay to evaluate the complement-mediated serum bactericidal activity of human sera against *S*. Typhimurium and *S*. Enteritidis by L-SBA, an anti-iNTS human standard serum was generated by pooling sera from highly positive subjects originally enrolled in the STRATAA epidemiological study, collected in Malawi, and tested against *S*. Typhimurium and *S*. Enteritidis strains, which were neat or diluted ad hoc to mimic samples with different bactericidal activity. In this work, the L-SBA is characterized in terms of acceptance criteria: definition of detection and quantification limits, repeatability, intermediate precision, linearity, and specificity. The aim of the study is to apply and refine the assay conditions already established at the preclinical level [23] to human sera and characterize the assay prior to testing the functionality of the antibodies from the clinical samples coming from Phase 1 studies to evaluate the safety, reactogenicity, and immune responses to an iNTS GMMA-based vaccine against both the *Salmonella* serovars Typhimurium and Enteritidis.

### 3.1. Precision

The precision of the method expresses the ability of a measurement to be consistently reproduced. It is considered at two levels: repeatability and intermediate precision. Repeatability expresses the precision under the same operating conditions and is also named intra-assay precision. Intermediate precision (occasionally called within-lab reproducibility) expresses within-laboratory variations, such as different days, analysts, equipment, calibrants, batches of reagents, columns, and spray needles. 

**Figure 1:**
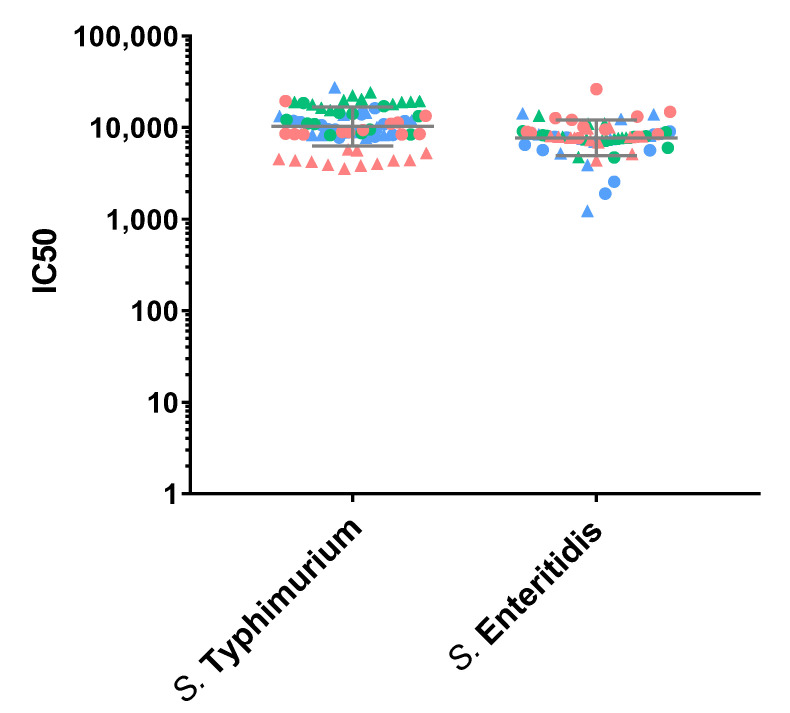
SBA titers (IC50) obtained from the precision test, performed by probing standard sera in 12 independent replicates, on 3 different days and by two operators against the *S*. Typhimurium D23580 and *S*. Enteritidis CMCC4314 strains. Single repeats of each operator are represented by circles (for operator 1) and triangles (for operator 2), and repeats on different days are shown in red for day 1, green for day 2, and blue for day 3. Geometric means and geometric standard deviations from all repeats are represented by the grey lines for each of the strains.

Thus, to assess precision of the assay, the IC50 for the iNTS Primary Human Standard Serum was determined independently by two operators, 12 times per day, and on three different days (72 measurements in total). Log-transformed IC50s obtained by both operators on each day are used to determine the repeatability and intermediate precision of the assay (Figure 1). The analysis against *S*. Typhimurium is characterized by an intermediate precision (CV% IP) of 5.74% and a repeatability (CV% R) of 2.67%. The analysis against *S*. Enteritidis is characterized by an intermediate precision (CV% IP) of 5.20% and a repeatability (CV% R) of 4.68%. The day and the operator, and also their interaction, resulted to be factors that did not influence assay variability (*p*-values > 0.05). The geometric means of IC50 from all the measurements are 11,495 for *S*. Typhimurium and 8414 for *S*. Enteritidis.

### 3.2. Linearity

To assess the linearity of the assay, the iNTS Primary Human Standard Serum was assayed neat and prediluted in PBS (4-fold, 8-fold, 16-fold, 32-fold, 64-fold, and 128-fold times) before being probed against the two *Salmonella* serovars in L-SBA. We considered the average of IC50 of the undiluted serum (obtained by precision analysis) as the nominal value, and from that one, we calculated the theoretical IC50 based on the dilutions by volume performed. The IC50 nominal values were then compared with the observed IC50 values obtained in the linearity analysis.

**Figure 2:**
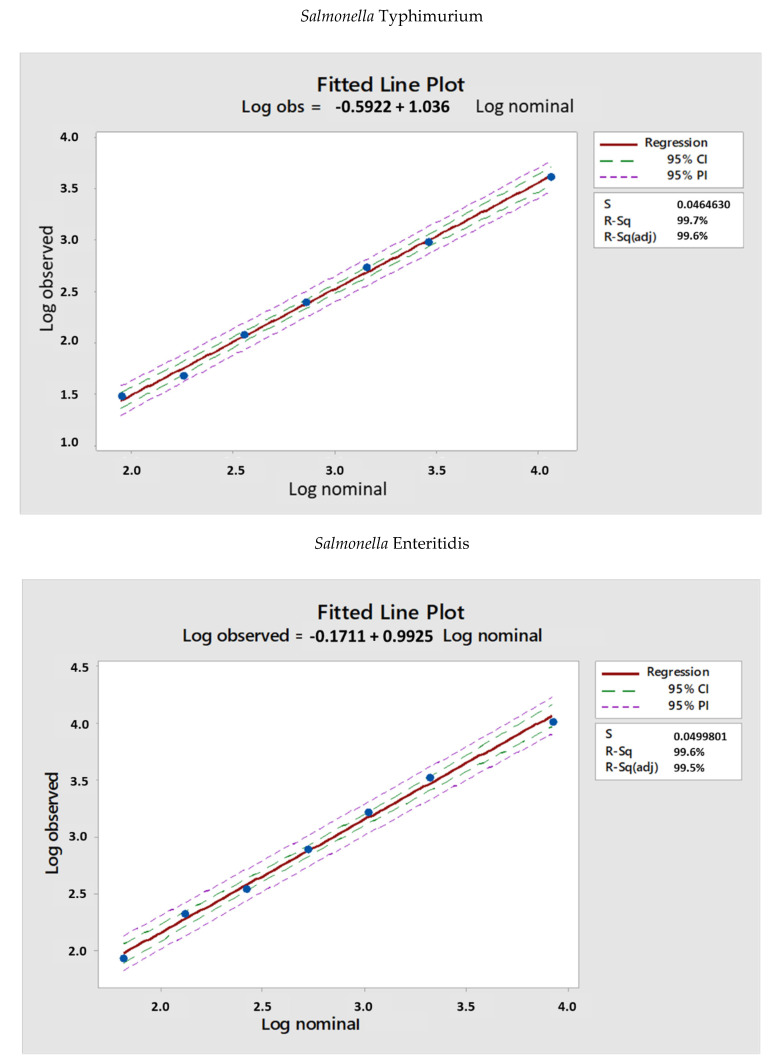
LogIC50 vs. log of serum dilution of samples tested to assess linearity both for the two *Salmonella* strains, *S*. Typhimurium D23580 and *S*. Enteritidis CMCC4314. Blue dots represent LogIC50 obtained for a specific serum diluted by each operator. The red line represents the linear regression trendline, and 95% CI (confidence interval) and 95% PI (prediction interval) are also reported.

The LogIC50 values obtained are reported in Figure 2, where experimental LogIC50 (observed) are plotted versus theoretical LogIC50 (nominal) and a regression analysis was performed in MiniTab. 

The two assays resulted to be linear, as the intercept was not significantly different from 0 and slope was not significantly different from 1.

### 3.3. Limit of Detection (LoD) and Limit of Quantification (LoQ)

**Figure 3:**
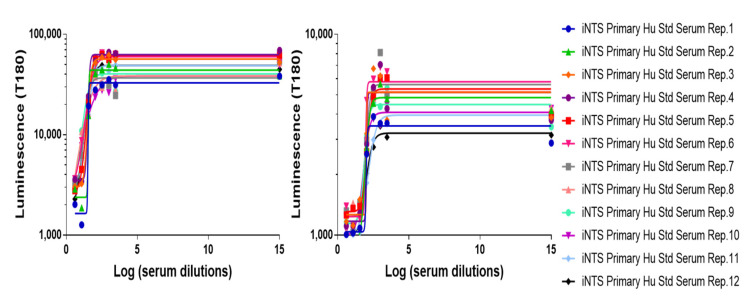
Luminescence vs. log of serum dilution of samples tested to assess *LoD* and *LoQ*. Symbols represent single measurements. Solid lines represent the fitted four-parameter curves to single-measurement data for each sample assayed against the two *Salmonella* strains, *S*. Typhimurium D23580 and *S*. Enteritidis CMCC4314.

The limit of detection (*LoD*) and the limit of quantification (*LoQ*) of the assay represent the lowest SBA titer that can be detected under the assay conditions and the lowest SBA titer that can be quantified with suitable precision, respectively. Thus, the HI iNTS Primary Human Standard Serum was prediluted 100-fold prior to being assayed in L-SBA against both *Salmonella*, *S*. Typhimurium D23580 and *S*. Enteritidis CMCC4314, in order to generate samples with low but detectable SBA titer. These conditions simulated a worst-case scenario for the assay, where it was expected to have the highest variability. Results obtained are reported in Figure 3.

Calculations of *LoD* and *LoQ* were performed according to the ICH guideline Q2(R1) (International Council for Harmonisation (ICH); ICH Q2(R1) Validation of Analytical Procedures: Text and Methodology; ICH: Geneva, Switzerland, 1995) by using the standard deviation (*SD*) of log-transformed SBA titers obtained for the samples and the lowest serum concentration tested in the assay (*X*, in our case = 4), according to the following formulas:

*LoD* = 10ˆ(3.3 * *SD*) * *X*

*LoQ* = 10ˆ(10 * *SD*) * *X*

*LoD* and *LoQ* resulted to be < 10 and < 55, respectively, with the IC50 obtained reported in Table 1.

### 3.4. Specificity

The specificity of the assay is the ability of an analytical procedure to determine solely the concentration of the analyte that it intends to measure.

To assess the homologous specificity, an initial setup experiment was performed by pre-incubating homologous *S*. Typhimurium and *S*. Enteritidis purified OAg at the final concentrations of 500, 200, 50, 10, and 1 µg/mL in PBS with test sera prior to performing the L-SBA. 

The goal was to determine the lowest concentration of OAg able to inhibit ≥ 70% of the IC50. However, this result was not reached at the conditions tested. Therefore, to better evaluate the specificity of the assay, we used the maximum OAg concentration possible for preincubation with human standard serum and also a different competitor, *Salmonella* GMMA purified from a homologous strain at 10, 50, 200, and 500 µg/mL. The non-depleted control was represented by HI iNTS Primary Human Standard Serum pre-incubated with an equal volume of PBS alone.

**Figure 4:**
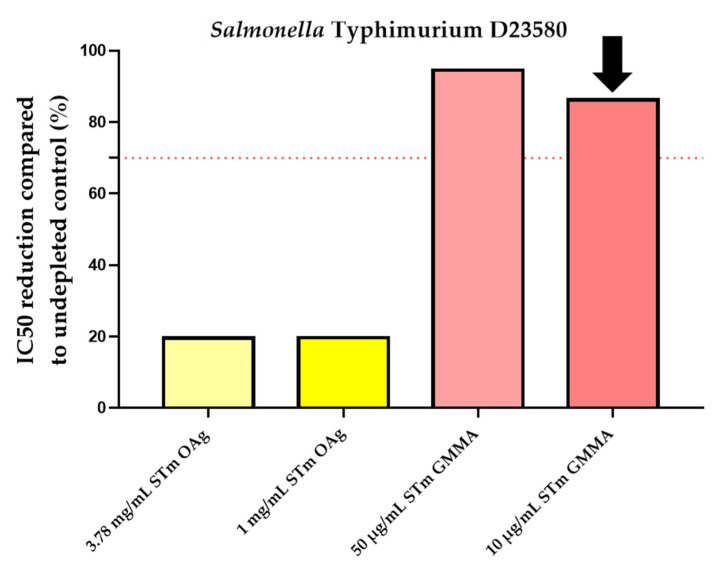

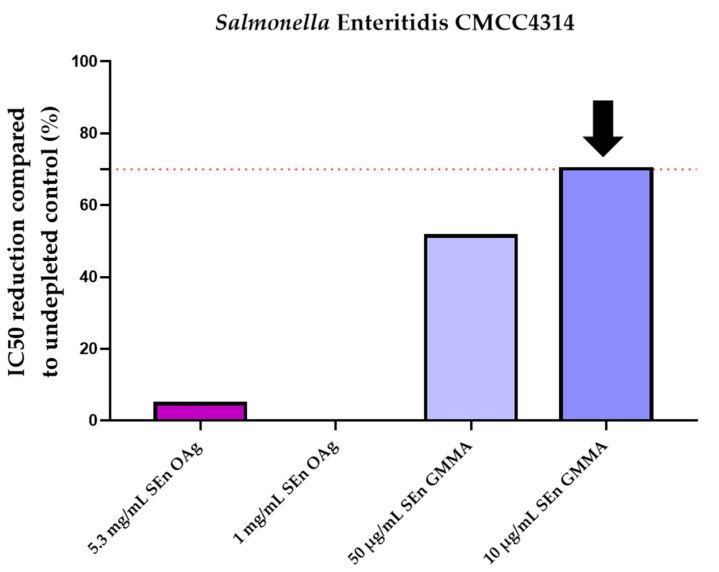
Homologous specificity for *Salmonella* strains: percentage of IC50 inhibition observed compared to control inhibited with equal amount of PBS. The lowest homologous *S*. Typhimurium and *S*. Enteritidis GMMA concentration that was able to inhibit ≥ 70% of the IC50 (indicated by the dotted red line) compared to the non-depleted control sample resulted to be 10 µg/mL, as indicated by the black arrows.

Results obtained showed an optimal inhibition (≥70%) only in the presence of *Salmonella* GMMA for both strains, as reported in Figure 4.

The percentage of inhibition was determined by calculating the decrease in the observed SBA titer between samples pre-treated with competitor and the undepleted control. The lowest homologous *S*. Typhimurium GMMA concentration (among the ones tested) that was able to inhibit ≥ 70% of the IC50 compared to the undepleted control sample was 10 µg/mL (86.2% depletion of SBA titer), as well as for *S*. Enteritidis (70.1% depletion of SBA titer), as reported in Table 2.

To determine the heterologous specificity, *S*. Typhimurium and *S*. Enteritidis (heterologous but from the same species) and *S. flexneri* 3a GMMA (heterologous from a different species) at the final concentration indicated in Table 3 (10 µg/mL) were incubated with an equal volume of the standard serum iNTS Primary Human Standard Serum. Internal controls for this experiment were represented by a sample preincubated with an equal volume of PBS alone (undepleted) and by a sample preincubated with homologous *Salmonella* GMMA (to confirm the homologous specificity observed in Figure 4). 

Specificity was determined as % IC50 inhibition; this was calculated using the following formula:

%IC50 inhibition = (IC50 of the undepleted sample)—(IC50 of the sample pretreated with competitor)/(IC50 of the undepleted sample) * 100

**Figure 5:**
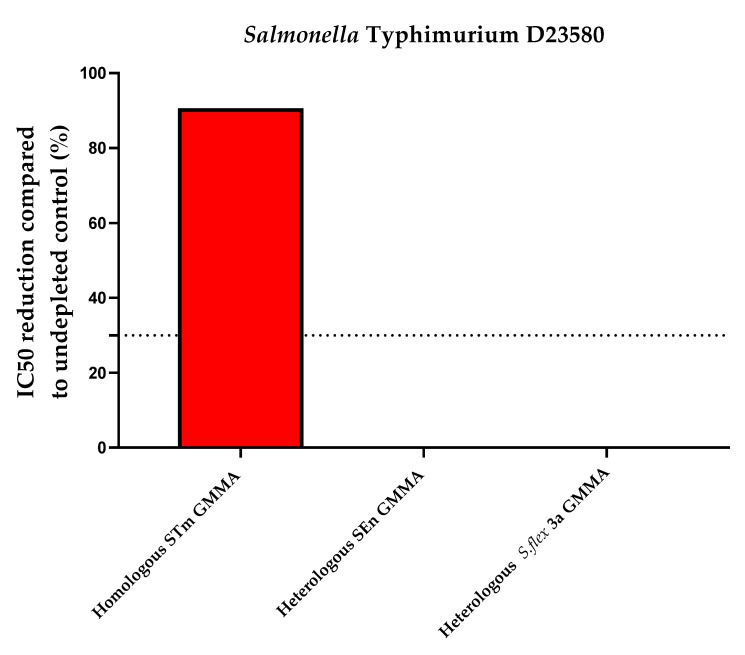

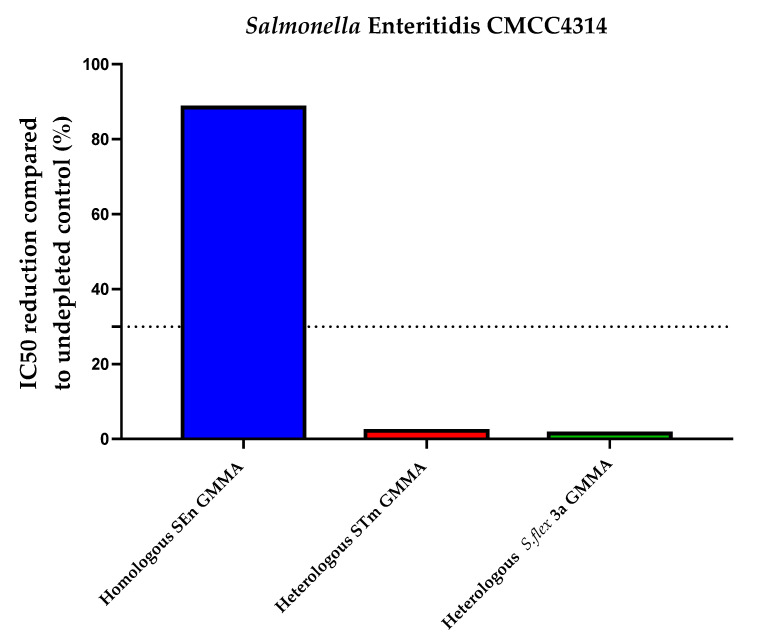
Heterologous specificity for *Salmonella* strains: percentage of IC50 inhibition observed compared to control inhibited with equal amount of PBS. Both assays resulted to be specific. STm: *S*. Typhimurium; SEn: *S*. Enteritidis; *S*. flex: *S. flexneri*.

Depletion with 10 µg/mL of *S*. Typhimurium and *S*. Enteritidis GMMA (homologous competitors for *S*. Typhimurium and *S*. Enteritidis, respectively) caused an inhibition of IC50 of 90.1% (*S*. Typhimurium) and 88.4% (*S*. Enteritidis), confirming the high specificity of the assay, whereas depletion with their heterologous GMMA resulted in an absent decrease in SBA titer, as indicated in Figure 5 and Table 3.

## 4. Discussion and Conclusions

Introduction of vaccines to control the high burden of disease caused by iNTS in sub-Saharan Africa is an urgent priority. To design optimal interventions, a full understanding of epidemiology is critical. Although no correlate of protection for *Salmonella* effectiveness is established, serum bactericidal assay (SBA) represents an in vitro method that has been widely used to evaluate the capability of antibodies present in serum to kill bacteria through complement activation [37]. Large sero-epidemiological studies in endemic countries, as well as vaccine clinical trials, would benefit from assays that are high-throughput, can be performed at a reasonable cost, and are not labor-intensive. Furthermore, to establish robust procedures, it is crucial to use standard sera, which can serve as calibrators for interlaboratory enzyme-linked-immunosorbent-assay (ELISA) serology analyses, as well as the same bacterial strains and critical reagents, like source of complement, commercially available. The traditional SBA method (CFU-based) had limitations in terms of time and operator dependency. To overcome these weaknesses, we developed a high-throughput SBA method based on a luminescent readout (L-SBA), both in 96-wells [29,30] and 384-wells [31] plate formats. Similar methodology has additionally been optimized and characterized to evaluate the functionality of antibodies elicited by a multivalent GMMA-based vaccine against *Shigella* on human sera, reported elsewhere [32].

In this work, we present the setup and the intra-laboratory characterization of iNTS L-SBA on human sera in terms of repeatability, intermediate precision, linearity, and specificity. We successfully optimized the fitting of the data by establishing multiple and solid acceptance criteria for analysis. We characterized L-SBA on human sera, demonstrating that, in the working conditions tested, the assay is able to detect sera with an SBA titer as little as 9.3 (in the case of *S*. Typhimurium) and 8.7 (in the case of *S*. Enteritidis), with theoretically no upper limit of detection, and to quantify with precision sera with an IC50 of 51.5 (in the case of *S*. Typhimurium) and 42.3 (in the case of *S*. Enteritidis). Both assays were demonstrated to be precise showing very low variability, with SBA against *S*. Typhimurium having an intermediate precision of 5.74% (CV% IP) and a repeatability of 2.67% (CV% R), whereas *S*. Enteritidis L-SBA was characterized by an intermediate precision (CV% IP) of 5.20% and a repeatability (CV% R) of 4.68%. Neither the operator nor the day of analysis nor their interaction was significant to the overall variability. Moreover, L-SBA was strongly specific for the key active ingredients of the vaccine candidate, as, by depleting the serum with as little as 10 µg/mL of homologous GMMA, more than 88% reduction of IC50 was observed, whereas no depletion was observed when depleting sera with heterologous OAg both from the same *Salmonella* species and from a different bacterial genus, *Shigella*. The linearity of the assay was also assessed and was found to be well maintained within the tested range. 

Since our L-SBA needs only a luminometer to detect ATP, the assay can be considered simple enough to be adopted by laboratories around the world, including endemic countries. The potential inter-laboratory variability could be overcome by normalizing the results with the use of a reference serum such as we created [38]. This is our next objective. The assay has been already demonstrated to have a similar performance against a broad range of pathogens using animal samples [29,30,39], and, in this work, as in a previous work on a *Shigella* GMMA-based vaccine [32], we conclude that, due to its high specificity and versatility and high throughput, L-SBA can be applied to determine the bactericidal activity of clinical sera raised against different *Salmonella* serotypes or from sero-epidemiological studies, supporting the development of vaccines to prevent iNTS disease. 

## Figures and Tables

**Table 1:** *LoD* and *LoQ*, expressed as IC50, for each of the two *Salmonella* strains tested, *S*. Typhimurium D23580 and *S*. Enteritidis CMCC4314.

	*S*. Typhimurium	*S*. Enteritidis
*LoD* (IC50)	9.3	8.7
*LoQ* (IC50)	51.3	42.3

**Table 2:** Minimum homologous GMMA concentration able to inhibit IC50 ≥ 70% for each of the two *Salmonella* strains tested, *S*. Typhimurium D23580 and *S*. Enteritidis CMCC4314.

SetUp of Minimum Homologous OAg Concentration to Inhibit IC50 ≥ 70%			
*S*. Typhimurium		*S*. Enteritidis	
Homologous GMMA concentration (µg/mL)	Percentage of inhibition compared to undepleted control	Homologous GMMA concentration (µg/mL)	Percentage of inhibition compared to undepleted control
10	86.2	10	70.1

**Table 3:** Percentage of IC50 depletion compared to undepleted control in presence of homologous and heterologous competitors at concentration established in the previous set of experiments.

Heterologous Specificity (Percentage of Inhibition Compared to Undepleted Control)					
*S*. Typhimurium			*S*. Enteritidis		
iNTS Primary Human Standard Serum depleted with 10 µg/mL STm GMMA	iNTS Primary Human Standard Serum depleted with 10 µg/mL SEn GMMA	iNTS Primary Human Standard Serum depleted with 10 µg/mL *S.flex* 3a GMMA	iNTS Primary Human Standard Serum depleted with 10 µg/mL SEn GMMA	iNTS Primary Human Standard Serum depleted with 10 µg/mL STm GMMA	iNTS Primary Human Standard Serum depleted with 10 µg/mL *S.flex* 3a GMMA
90.1	0	0	88.4	2.2	1.5

STm: *S*. Typhimurium; SEn: *S*. Enteritidis; S. flex: *S. flexneri*.

## Data Availability

Not Applicable.
